# Fixation versus primary replacement of displaced femoral neck fractures in the elderly

**DOI:** 10.4103/0019-5413.73658

**Published:** 2011

**Authors:** Moin Khan, Ilyas S Aleem, Rudolf W Poolman

**Affiliations:** McMaster University, Hamilton, Ontario; 1 University of Toronto, Toronto, Ontario, Canada; 2 Onze Lieve Vrouwe Gasthuis, Department of Orthopedic Surgery, Joint Research, Amsterdam, The Netherlands

**Keywords:** Arthroplasty, controversies in fixation, hip fractures

## Abstract

Hip fractures are among the most common debilitating injuries in the elderly and are a significant cause of morbidity and mortality worldwide. Despite the ever-increasing literature on the topic of hip fractures, optimal treatment remains uncertain. Trials with small sizes, methodological limitations, strict inclusion criteria and wide confidence intervals leave the optimal approach to treating hip fractures unknown and controversial. In 2005, the International Hip Fracture Research Collaborative was officially established with the mandate of resolving controversies in hip fracture management. Presently, two multicenter randomized trials, FAITH and HEALTH, are underway. The FAITH trial (Fixation Using Alternative Implants for the Treatment of Hip Fractures) will compare Sliding Hip Screws and Cancellous Screws; the HEALTH trial (Hip Fracture Evaluation with Alternatives of Total Hip Arthroplasty versus Hemi-Arthroplasty) will compare total hip arthroplasty and hemi-arthroplasty. The present paper reviews current controversies in hip fracture care. Ultimately, only large randomized trials, such as FAITH and HEALTH, will resolve the longstanding controversy of whether primary replacement or fixation is the preferred treatment modality in this common fracture. Subsequent trials need to focus on surgical strategies in the cognitively impaired patient.

## INTRODUCTION

Hip fractures are among the most common fractures in the elderly and are a significant cause of morbidity and mortality in the age group 65 years and older.^1^,^2^ Disability associated with hip fractures in the elderly imposes an increasing burden on the healthcare system, globally creating a need for high-quality research to advance the care of these patients.^3^ By the year 2040 it is anticipated that the number of hip fractures in the United States will exceed 500000 annually with an estimated annual healthcare cost of over US$9.8 billion.^4^ In India alone there is an estimated 440000 hip fractures each year, a number that is expected to rise to 600000 by 2020 and more than 1 million by 2050.^5^ Osteoporosis is more widespread in this region in comparison to their North American counterparts due to vitamin D deficiency, poor nutrition and smaller skeletal size.^5^

The treatment goal in the management of these fractures is to bring patients back to their pre-morbid level of activity and functional status. Therefore, in order to improve outcome in patients following hip fracture it is essential to determine the optimal method of treatment for a particular patient. When deciding on a definitive operative plan, surgeons must look at their individual patient population and take into consideration a number of factors including health status of patients, age, functional and cognitive limitations, bone quality, and individual patient goals and expectations.^6^ Despite the ever-increasing literature on hip fracture management the optimal treatment remains unknown. The purpose of this review is to include a discussion on the current controversies regarding treatment options for displaced femoral neck fractures in the elderly as well as upcoming research in the field aimed at providing the data needed to determine optimal care for this common injury.

## TREATMENT OPTIONS

Treatment alternatives for displaced femoral neck fractures include arthroplasty and internal fixation. Options for arthroplasty include total hip arthroplasty and hemi-arthroplasty (HA); options for internal fixation include multiple screws and sliding hip screw fixation. Surveys of orthopedic surgeons have demonstrated varying practices in the treatment of such fractures. In a study by Chua *et al*., (1997), there was virtually a 50–50 split with the surgeons surveyed regarding the treatment of an independent 70-year-old woman with no co-morbidities with regards to hemi-arthroplasty or internal fixation.^7^

In comparison to internal fixation, proponents of arthroplasty cite higher levels of postoperative function^7^ and a lower need for reoperation as replacement of the femoral head eliminates the risk for avascular necrosis and nonunion.^1^,^7^,^9^ In a trial of 222 patients, Frihagen *et al*., (2007), demonstrated that when compared to internal fixation, hemi-arthroplasty results in better hip function, higher health-related quality of life, and more independence. Bhandari *et al*., (2003), showed that the outcome of displaced femoral neck fractures treated with internal fixation required reoperation in 35% of 1901 patients. Advantages of hemi-arthroplasty over total hip replacement are simple faster surgical technique, lower risk of dislocation, and short-term economic benefits.^10^,^11^ Disadvantages of HA mainly consist of rapid wear of acetabular articular cartilage and pain related to the femoral head against the acetabulum.^12^,^13^ Advantages of total hip replacement compared to HA are superior and more durable function and possible economic benefits with respect to long-term cost of treating failures of HA and internal fixation.^10^,^11^,^14^–^16^ Surgeons favoring internal fixation prefer the shorter operative time, decreased blood loss, decreased risk of dislocation and reduced risk of postoperative wound infection.^1^,^13^,^17^–^19^ A recent long-term prospective multicenter study by Leonardsson *et al*., (2010), involving 450 patients over the age of 70 randomized to either internal fixation or replacement found that at 10 years postoperatively failure rate with internal fixation (45.6%) was greater than replacement (8.8%). The most common causes for this being avascular necrosis and nonunion. There were no differences with regards to mortality in either population. Patient-reported pain and function was similar at the five and 10-year mark in both groups. The study also found that replacement was not associated with excess complications such as aseptic loosening or periprosthetic fractures within 10 years.^20^

## CONTROVERSIES IN HIP FRACTURE CARE AND EVIDENCE-BASED ORTHOPEDICS

Despite the ever-increasing literature on the topic of hip fractures, the ideal treatment option remains unknown. Trials with small sample sizes, methodological limitations and wide confidence intervals leave much uncertainty.^3^ Such trials have been common in orthopedic literature. In the Journal of Bone and Joint Surgery, American Volume, from January 2003 to December 2004 only 3.4% were randomized control trials.^21^ Randomized control trial sizes have ranged from 18 to 552 participants with the average sample size being 113 ± 102 participants. Furthermore, 77% of the studies were single-center initiatives and only one-third of the trials performed an a prior sample size calculation.^22^ The quality of the data is also negatively affected by issues such as unclear allocation concealment, no mention of surgeon skill or experience and assessor bias. Intention to treat analysis was judged to be used in 44% of studies.^22^

Controversies in hip fracture care have resulted in many debates in international orthopedic surgical meetings. The Orthopedic Trauma Association (OTA), Canadian Orthopedic Association (COA), The American Academy of Orthopedic Surgeons (AAOS) and the International Society for Fracture Repair (ISFR) have all presented Symposia discussing the importance of the issue.^3^ In a survey of 298 North American and European orthopedic surgeons treating patients aged 65-80 years old with displaced hip fractures, variability in surgeons’ preferences for management was identified with regards to arthroplasty as well as internal fixation.^7^ The goal is a need for large, international, rigorously performed randomized control trials (RCTs) with sufficient power to determine the optimal approach to treating hip fractures. Only large, rigorously performed randomized trials will demonstrate the optimal type of internal fixation, the superior form of arthroplasty and finally whether arthroplasty or internal fixation is the preferred treatment option.^3^,^7^,^23^

Current evidence-based practice emphasizes the importance of randomized clinical trials when possible. When looking at large multicenter RCTs in other medical areas, we see 20 years ago the management of acute coronary syndromes was uncertain. Large international randomized trials, however, led to an enormous reduction in mortality and morbidity through demonstration of optimal management approaches.^3^ Recent symposia have discussed the importance of specialty societies to facilitate large-scale randomized clinical trials.^24^

## INTERNATIONAL HIP FRACTURE RESEARCH COLLABORATIVE

In 2005 the International Hip Fracture Research Collaborative (IHFRC) was officially established with the mandate of resolving controversies in hip fracture management.^3^ The aims of the group are: 1) to identify key unresolved issues and focus future clinical research in the operative management of patients with hip fractures; 2) to bridge smaller ongoing research networks in North America and Europe into a large, single collaborative effort; and 3) to design, plan and coordinate timely large randomized trials to provide definitive answers to the priority research questions identified by the participating investigators.

## FAITH AND HEALTH TRIALS

Presently, two large multicenter randomized trials, FAITH and HEALTH are underway.^3^ The FAITH trial (Fixation Using Alternative Implants for the Treatment of Hip Fractures) is a multicenter randomized trial comparing sliding hip screws and cancellous screws on revision surgery rates in the treatment of femoral neck fractures. The HEALTH trial (Hip Fracture Evaluation with Alternatives of Total Hip Arthroplasty versus Hemi-arthroplasty) is a multicenter randomized trial comparing total hip arthroplasty and hemi-arthroplasty in patients with femoral neck fractures. The final study after completion of the FAITH and HEALTH trials will compare the optimal internal fixation versus optimal approach to arthroplasty.

Effective infrastructure is essential for clinical trials to be successful. At McMaster University (Hamilton, ON) a Methods Center has been established to coordinate the FAITH and HEALTH trials. Country offices have also been established for the facilitation of communication between the Methods Center and the clinical sites in the participating countries where language and communication barriers are likely to exist. This will ensure that the trial protocol is followed and the data that is collected is accurate and complete.

## ARE THE RESULTS APPLICABLE TO ALL PATIENTS?

Previous studies have excluded patients with dementia while others have focused on this complex patient group.^8^,^15^,^25^,^26^ Including or excluding cognitively impaired patients complicates decision-making based on results from these trials. For example, the RCT by Keating *et al*., (2006) excluded 803 patients (30% of patients screened) based on a failed mental test. Frihagen *et al*.,(2007) included both patients with previously recognized cognitive failure and mentally fit individuals. A subgroup analysis was not conducted by these investigators for patients with dementia making inferences less direct.^8^ Frihagen *et al*., found superior results for hemi-arthroplasty based on less complications and better functional outcome. However, in a small RCT with 60 patients, van Dortmont *et al*., found that hemi-arthroplasty was associated with significantly more loss of blood and increased wound complications. These conflicting results stress the need for sufficiently powered studies focusing on patient outcomes in cognitively impaired individuals. Moreover, economic analyses need to be performed given the increased burden for society caring for patients with dementia. Although HEALTH and FAITH will help in finding answers, applicability to all hip fracture patients remains a challenge. The current trials exclude cognitively impaired patients but can aid in framing future research questions. Subsequent studies need to be designed including, or specifically focusing on, this complex and increasing patient population.

## CONCLUSIONS

Randomized trials should play an important role in determining the best practice to care for patients with hip fractures. The current data is inconclusive on the optimal approach to treating these fractures. Ultimately, only large, international, rigorously tested randomized trials such as FAITH and HEALTH will result in improvements in the outcomes of treatment and resolve the longstanding controversy of whether primary replacement or fixation is the preferred treatment modality for hip fractures. Subsequent trials need to focus on surgical strategies in the cognitively impaired patient.
